# Postoperative Discharge Destination Impacts 30-Day Outcomes: A National Surgical Quality Improvement Program Multi-Specialty Surgical Cohort Analysis

**DOI:** 10.3390/jcm12216784

**Published:** 2023-10-26

**Authors:** Carlos Riveros, Sanjana Ranganathan, Yash B. Shah, Emily Huang, Jiaqiong Xu, Michael Geng, Zachary Melchiode, Siqi Hu, Brian J. Miles, Nestor Esnaola, Dharam Kaushik, Angela Jerath, Christopher J. D. Wallis, Raj Satkunasivam

**Affiliations:** 1 Department of Urology, Houston Methodist Hospital, Houston, TX 77030, USA; cariveross1@gmail.com (C.R.); ranganathan.sanjana@gmail.com (S.R.); emily.you.huang@gmail.com (E.H.); zsmelchiode@houstonmethodist.org (Z.M.); shu@houstonmethodist.org (S.H.); bjmiles@houstonmethodist.org (B.J.M.); dkaushik@houstonmethodist.org (D.K.); 2 Sidney Kimmel Medical College, Thomas Jefferson University, Philadelphia, PA 19107, USA; yash.shah2@students.jefferson.edu; 3 Center for Health Data Science and Analytics, Houston Methodist Research Institute, Houston, TX 77030, USA; sxu@houstonmethodist.org; 4 School of Engineering Medicine, Texas A&M University, Houston, TX 77030, USA; michael.j.geng@gmail.com; 5 Department of Surgery, Houston Methodist Hospital, Houston, TX 77030, USA; nfesnaola@houstonmethodist.org; 6 Department of Anesthesia, Sunnybrook Health Sciences Center, Toronto, ON M4N 3M5, Canada; angela.jerath@sunnybrook.ca; 7 Division of Urology and Surgical Oncology, Department of Surgery, Princess Margaret Cancer Centre, University Health Network, University of Toronto, Toronto, ON M5R 0A3, Canada; wallis.cjd@gmail.com; 8 Division of Urology, University of Toronto, Toronto, ON M5R 0A3, Canada; 9 Division of Urology, Mount Sinai Hospital, Toronto, ON M5G 1X5, Canada

**Keywords:** discharge, surgery, outcomes, destination, readmission, complications

## Abstract

Surgical patients can be discharged to a variety of facilities which vary widely in intensity of care. Postoperative readmissions have been found to be more strongly associated with post-discharge events than pre-discharge complications, indicating the importance of discharge destination. We sought to evaluate the association between discharge destination and 30-day outcomes. A retrospective cohort study was conducted using the American College of Surgeons National Surgical Quality Improvement Program (ACS-NSQIP) database. Patients were dichotomized based on discharge destination: home versus non-home. The main outcome of interest was 30-day unplanned readmission. The secondary outcomes included post-discharge pulmonary, infectious, thromboembolic, and bleeding complications, as well as death. In this cohort study of over 1.5 million patients undergoing common surgical procedures across eight surgical specialties, we found non-home discharge to be associated with adverse 30-day post-operative outcomes, namely, unplanned readmissions, post-discharge pulmonary, infectious, thromboembolic, and bleeding complications, as well as death. Non-home discharge is associated with worse 30-day outcomes among patients undergoing common surgical procedures. Patients and caregivers should be counseled regarding discharge destination, as non-home discharge is associated with adverse post-operative outcomes.

## 1. Introduction

Unplanned 30-day readmission rates are a prevailing quality metric in evaluating hospital performance [1]. Currently, 30-day readmissions occur in approximately 15% of Medicare surgical discharges, and 90% of these are unplanned [2]. In 2018, there were 3.8 million readmissions, with each costing an average of USD 15,200 [3]. Per the value-based Hospital Readmissions Reduction Program (HRRP) Medicare initiative, hospitals are penalized based on their unplanned 30-day readmission rates [4]. Although hospital care significantly impacts readmissions among postoperative patients, numerous biologic, healthcare, and social factors outside the hospital itself may also play a role [5].

In addition to their homes, patients can be discharged to a variety of facilities which vary widely in level of care [6]. In 2015, Merkow et al. characterized the underlying reasons associated with unplanned hospital readmissions for postoperative patients. Readmissions were more strongly associated with post-discharge events than pre-discharge complications, indicating the importance of discharge destination. Although they found that non-home discharge was associated with 1.4 times greater odds of unplanned readmission, they examined only six surgical procedures and noted that this may not be representative of all operations [7].

Given the latest shifts in insurance coverage and healthcare infrastructure, an updated analysis is necessary to further elucidate the impact of discharge destination in a nationally representative cohort. We sought to investigate differences in readmission rates and post-discharge complications based on discharge destination using a multi-specialty surgical cohort containing over 1.5 million patients in the United States. We hypothesized that home discharge would be associated with a lower risk of unplanned readmission and other post-discharge complications.

## 2. Materials and Methods

### 2.1. Data Source

This retrospective study utilized data from the American College of Surgeons National Surgical Quality Improvement Program (ACS-NSQIP), a nationally validated, risk-adjusted, and prospectively collected surgical database. The NSQIP employs designated clinically trained abstractors across nearly 700 participating hospitals to record 30-day perioperative data and outcomes [8]. As the database contains deidentified data, this study was deemed exempt by our Institutional Review Board. This study was reported according to the Strengthening the Reporting of Observational Studies in Epidemiology (STROBE) reporting guidelines and the Reporting of Studies Conducted Using Observational Routinely-Collected Health Data (RECORD) statement [9,10].

### 2.2. Cohort Selection

We included adult patients (≥18 years old) who had undergone common surgical procedures across multiple surgical subspecialties between 2005 and 2020. After consulting a multidisciplinary team of subspecialists, a consensus decision-making approach was utilized to generate a multiprocedural cohort with a high degree of generalizability. Using Common Procedural Terminology (CPT) codes, we identified representative major surgical procedures across eight different surgical subspecialties (gynecology, neurosurgery, orthopedics, urology, thoracic, general, cardiac, and vascular surgery; Supplementary Table S1). We excluded patients not admitted from home, those who died during the index hospital stay, and those discharged to a hospice or against medical advice. Patients with a missing body mass index (BMI), American Society of Anesthesiologists (ASA) status, preoperative laboratory values, operative time, length of stay, discharge destination, and readmission status were also excluded.

### 2.3. Outcome

The primary outcome of interest was unplanned readmission, defined as a readmission after discharge from the index hospital stay and within 30 days of the primary surgical procedure. The readmission must have constituted a hospital stay of at least two midnights and could have been to the index hospital or any other institution. Transfers from the index hospital to another acute care facility are not counted as readmissions [11]. Secondary outcomes included post-discharge pulmonary (prolonged intubation or reintubation), infectious (surgical site infections, pneumonia, urinary tract infection, or sepsis), thromboembolic (deep venous thrombosis and pulmonary embolism), and bleeding complications, as well as death.

### 2.4. Exposure

The exposure of interest was discharge destination, dichotomized as home or non-home. Non-home discharge was defined as discharge to a skilled care facility (e.g., a transitional care unit, subacute hospital, or skilled nursing home), unskilled care (e.g., nursing home or assisted facility, only if this was not the patient’s preoperative location), separate acute care, rehabilitation, or a multi-level senior community.

### 2.5. Covariates

Relevant covariates included age, sex, race/ethnicity, BMI, hypertension, diabetes mellitus, congestive heart failure, chronic obstructive pulmonary disease, dyspnea, ascites, bleeding disorder, chronic steroid use, weight loss > 10% in the last six months, chronic kidney disease, smoking history, ASA status, functional status, preoperative hematocrit, platelet and white blood cell count, surgical subspecialty, operative time, length of index hospital stay, and major pre-discharge complications. Body mass index was classified into underweight, normal, overweight, and class I, II, and III obesity [12]. The estimated glomerular filtration rate was calculated using the Chronic Kidney Disease Epidemiology Collaboration (CKD-EPI) equation [13]. Chronic kidney disease was classified using the 2012 Kidney Disease: Improving Global Outcomes (KDIGO) guidelines [14]. Major pre-discharge complications were defined as: unplanned reoperation, cardiac arrest, myocardial infarction, or stroke occurring before discharge [15].

### 2.6. Statistical Analysis

Continuous variables were reported using mean and standard deviation (SD) or median and interquartile range (IQR), while categorical variables were reported using frequency and proportions. A logistic regression model was fitted to discharge destination as the outcome, while the covariates used were age, sex, race/ethnicity, BMI, hypertension, diabetes mellitus, congestive heart failure, chronic obstructive pulmonary disease, dyspnea, ascites, bleeding disorder, chronic steroid use, weight loss > 10% in the last six months, chronic kidney disease, smoking history, ASA status, functional status, preoperative hematocrit, platelet and white blood cell count, surgical subspecialty, operative time, length of index hospital stay, and major pre-discharge complications.

Using the results of the model, 1:1 propensity score matching (PSM) was performed using the nearest-neighbor method and a caliper size of 0.2 to identify the impact of discharge destination on 30-day unplanned readmissions and post-discharge complication rates. The standardized mean difference (SMD) was measured to determine the balance between the two groups before and after PSM. An absolute SMD value < 0.1 was used as the cutoff for sufficient balance between the two groups. Thirty-day unplanned readmission rates were calculated for each discharge destination group using Kaplan–Meier estimates after PSM. Conditional logistic regression models were used to adjust for those factors that remained unbalanced after PSM and to account for the clustering created through matching. Odds ratios (OR) and their 95% confidence intervals (CI) were reported as a measure of association between discharge destination and each outcome of interest.

We performed a sensitivity analysis using the E-value, which represents the minimum strength of association that an unmeasured confounder must have with both the exposure and outcome to fully explain away a specific association, conditional on the measured covariates [16]. Essentially, a higher E-value indicates that any unmeasured confounder must be proportionally stronger to explain the observed association [17]. All reported *p*-values were two-sided with the significance level at 0.05. All analyses were performed with STATA 16.1 (StataCorp. 2019. Stata Statistical Software: Release 16. College Station, TX, USA: StataCorp LLC).

## 3. Results

### 3.1. Patient Demographics and Outcomes

**Figure 1:**
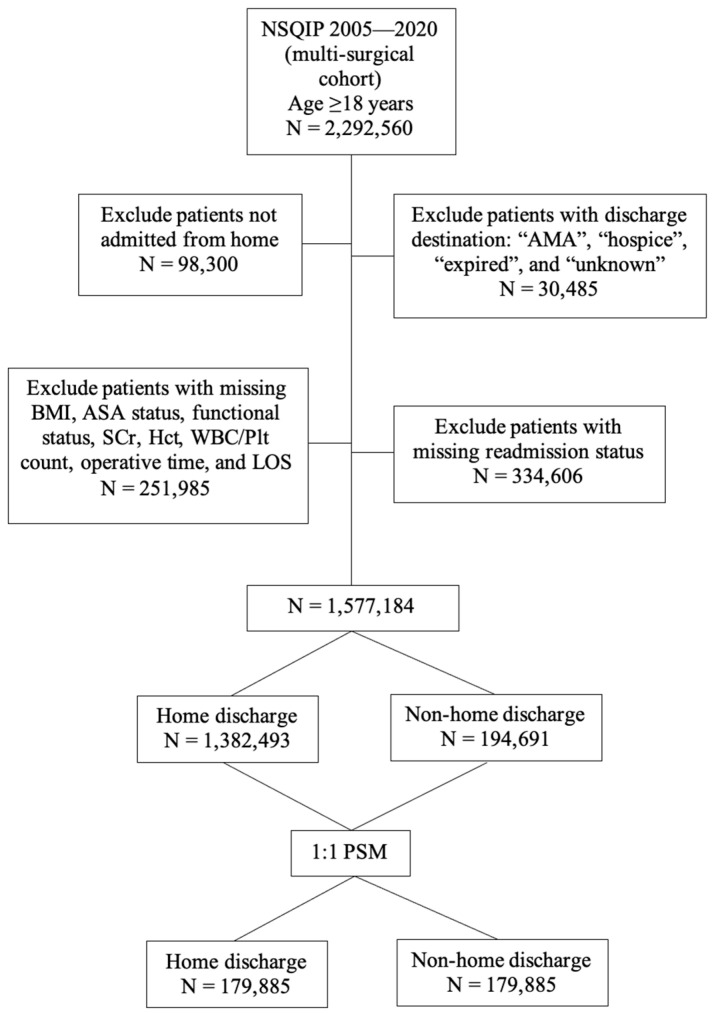
Flowchart for patient selection. NSQIP: National Surgical Quality Improvement Program; AMA: against medical advice; BMI: body mass index; ASA: American Society of Anesthesiologists; SCr: serum creatinine; Hct: hematocrit; WBC: white blood cell; Plt: platelet; LOS: length of stay; PSM: propensity score matching.

The final cohort consisted of 1,577,184 patients, with 1,382,493 (87.7%) and 194,691 (12.3%) patients discharged to home and non-home destinations, respectively (Figure 1). The mean age of the study cohort was 62.03 years, with 60.0% female, 71.8% White, and 79.2% non-Hispanic patients. Demographics and baseline comorbidities were compared between the two discharge destination groups (Table 1). An imbalance was identified in nearly all the compared variables except sex, BMI, smoking status, ascites history, chronic steroid use, recent weight loss > 10%, disseminated cancer history, and preoperative platelet counts. Most notably, those discharged to home were significantly younger (mean age: 60.7 vs. 71.2 years). The proportion of patients with an independent functional status was significantly higher in patients discharged to home (98.9% vs. 92.7%). Moreover, patients discharged to home were less likely to have comorbidities including hypertension (52.4% vs. 72.9%), diabetes (16.8% vs. 27.5%), COPD (4.0% vs. 9.0%), and higher-stage CKD (Stage 5: 0.6% vs. 3.0%). The proportion of patients with a high ASA status was also significantly lower in patients discharged to home (ASA 4: 5.0% vs. 15.4%). Discharge destination selection appeared to vary according to surgical subspecialty. Orthopedic surgery, neurosurgery, and cardiac surgery specialists were significantly more likely to discharge patients to non-home sites, unlike vascular, general, urologic, thoracic, or gynecologic surgeries.

### 3.2. Patient Characteristics Stratified by Discharge Destination

Propensity score matching yielded 179,885 matched pairs in each group (Table 2). Except for the length of stay, all SMDs were below 0.1, indicating that the covariates were appropriately balanced between the two groups.

### 3.3. Readmission Rates and Post-Discharge Complications Stratified by Discharge Destination after PSM

Non-home discharge was found to be strongly associated with increased rates of several post-discharge adverse events (Table 3). Specifically, unplanned readmissions occurred in 9.3% of non-home discharge versus 7.3% of home discharge patients (OR 1.27, 95% CI 1.23*–*1.30). Moreover, post-discharge pulmonary (OR 2.63, 95% CI 2.33*–*3.03) and infectious complications (OR 1.37, 95% CI 1.32*–*1.41) occurred at significantly higher rates among non-home discharge patients. VTE rates (OR 1.52, 95% CI 1.41*–*1.61) and bleeding events requiring transfusion were also reported at higher rates (OR 2.56, 95% CI 2.22*–*2.94). Finally, the risk of death was significantly higher for patients discharged to home (OR 2.38, 95% CI 2.17*–*2.63).

The results of the sensitivity analyses conducted using E-values are shown in Table 3, alongside the ORs for each outcome. The E-values for the effect estimate and the upper CI limit of 30-day unplanned readmission were 1.85 and 1.77, respectively. In other words, an unmeasured confounder would need to be associated with both the non-home discharge destination and 30-day unplanned readmission based on ORs of 1.85 each, above and beyond the measured covariates, to fully explain away the observed association between the two variables.

**Figure 2:**
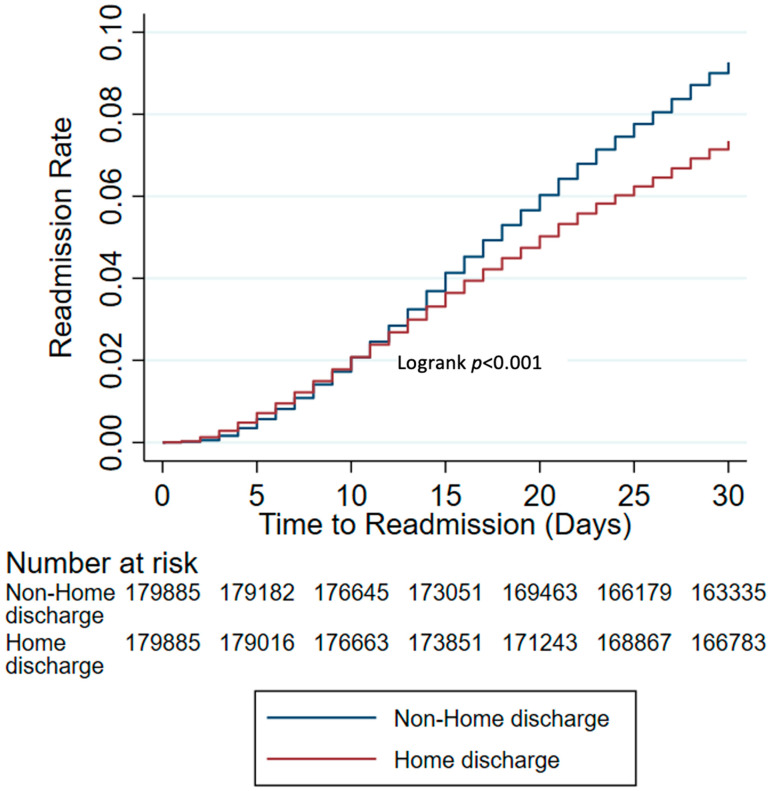
Readmission rates according to discharge destination after propensity score matching.

The Kaplan–Meier estimates showed that readmission rates were higher for patients who were discharged to non-home locations (Figure 2). This trend became more noticeable as time post-discharge increased, with a separation most clearly occurring after day 15. At 30 days postoperatively, the non-home discharge cohort appeared to have an almost 30% increase in their readmissions rates (9.3% vs. 7.3%, *p* < 0.001).

## 4. Discussion

In a nationally representative, multi-procedural cohort of over 1.5 million patients, we found that non-home discharge was associated with a greater risk of unplanned readmission, post-discharge pulmonary, infectious, VTE, and bleeding complications, as well as death. These outcomes were compared between discharge destinations after propensity score matching, a robust technique used to reduce the risk of confounding inherent in patients that are discharged to non-home destinations. While we hypothesized that being sent to post-acute care facilities may reduce the risk of complications, the opposite effect was observed. This is counterintuitive and may be explained by unmeasured confounders among non-home discharge patients.

Our sensitivity analyses (E-values with their corresponding CI limits) suggest the unmeasured confounder(s) would need to be of much greater magnitude to explain away the effect estimates revealed through the primary analyses. In line with the recommended guidelines for the use of E-values [18], potential unmeasured confounders in our study include social determinants of health, such as the level of social deprivation and the extent of support networks [19,20]. For instance, high social deprivation has been shown to be associated with non-home discharge following total hip arthroplasty among patients <65 (OR 1.47, 95% CI 1.34–1.61) and ≥65 years old (OR 1.31, 95% CI 1.22–1.41) [21]. High social deprivation has also been shown to be associated with any NSQIP complication following surgery (OR 1.11, 95% CI 1.02–1.22). Despite the use of PSM, the possibility of residual confounding remains within variables that are not captured in the NSQIP database. To the best of our knowledge, we are unaware of any unmeasured confounder(s) between the discharge destination and each of our outcomes of interest strong enough to explain away the observed associations.

Other studies support the notion of an increased risk of adverse outcomes with non-home discharge, albeit in smaller samples often limited by surgical subspecialty or procedure. Non-home discharge has been linked with higher readmission rates [7,22,23,24,25], alongside major complications, minor adverse events, and reoperations [26]. Non-home discharge has been demonstrated to be a predictor of mortality [27]. However, these findings are not consistent, as other studies have been inconclusive or contradictory [28,29,30].

Our findings both corroborate previous literature and provide new insights into quality improvement. Comorbidity analysis has shown that home discharge is more commonly assigned to younger and healthier patients [28,29,30]. This is logically sound, as providers recommend non-home discharge to presumably provide augmented care for at-risk patients, such as greater nursing and physiotherapy interventions. However, despite adjustment for this selection bias via PSM, our findings demonstrate that non-home discharge patients experience poorer outcomes. These distinct findings raise concerns, as assumptions that the higher level of care at non-home discharge sites will benefit at-risk patients are often used to justify the greater financial cost. The yearly expenditure on post-acute care exceeds USD 60 billion in Medicare alone [31]. However, there is little evidence to suggest that post-acute care leads to improved patient outcomes [32]. In fact, almost a quarter of Medicare beneficiaries experience adverse events during their stays at post-acute care facilities [33]. Preventable adverse events include nosocomial infections, falls, and medication errors [29,34].

Post-acute care facilities should technically provide increased surveillance and options to treat minor issues in-house, hence reducing costly discretionary readmissions. Our study and others demonstrate a possible failure-to-rescue pattern among these facilities, particularly for serious issues [30,35]. Notably, non-home discharge patients have been shown to have fewer readmissions for failure to thrive, nausea or vomiting, and dehydration, indicating that post-acute care facilities may reduce readmission rates for minor complications [28].

Given the failure to improve outcomes through the use of non-home discharge facilities, our study indicates a need for better risk approximation to ensure that patients are only assigned non-home disposition when necessary. Home discharge certainly improves economic measures, independence, and quality of life, and our study indicates that it may also improve postoperative safety. Patient-specific decision making is vital, and the Surgical Risk Preoperative Assessment System (SURPAS) and ACS-NSQIP risk calculator are parsimonious tools which predict postoperative complications. The Skilled Nursing Facility Readmission Risk (SNFRR) instrument evaluates the risk of 30-day hospital readmission among patients discharged to SNFs [36]. Mean 30-day, all-cause, standardized costs have been found to be positively associated with SNFRR score quartiles, with higher costs among patients at higher risk of readmission [37]. Each of these tools could be added to institutional electronic health record programs to improve quality metrics [38,39]. Further directions to improve outcomes and predict readmission may revolve around the use of biomarkers [40].

Given the benefits of home discharge, there are several additional options to further facilitate safety at home and therefore reduce the need for non-home discharge. Specifically, scheduled telephone and home visits, early surgeon or primary care provider follow-up appointments, and improved patient education surrounding post-operative care and self-monitoring have demonstrably reduced readmissions [27,39]. Improved coordination with the outpatient provider, hence reducing care fragmentation, is essential [7]. These interventions have improved value on the institutional level [41].

The literature has shown disparities in discharge determinations, and our analysis suggests that these differences may lead to tangible differences in health outcomes, hence further substantiating the need to improve discharge disposition decision making. Specifically, patients identifying as Black, living in communities scoring higher on the Community Deprivation Index, and those of lower socioeconomic status are more commonly placed in non-home destinations [21,42,43]. Similarly, high-volume hospitals, which have been shown to have better outcomes, were two times more likely to discharge patients to home. This was due in part to the higher utilization of home health services among high-volume hospitals, even in patients having postoperative complications [44].

Despite the valuable insights provided by our study, its limitations must be noted. Importantly, the NSQIP database may not capture certain comorbidities which may influence outcomes. Among patients discharged home, we did not have data regarding the use of home health services, as the NSQIP does not make that distinction. The number of non-home discharges included in the NSQIP is low, precluding stratification by type of non-home discharge facility (e.g., unskilled vs. skilled nursing facilities). The category, size, and economic factors of each facility may impact patient outcomes. We accounted for these limitations using a novel sensitivity analysis, which demonstrated that unmeasured confounders are unlikely to explain away our findings. Furthermore, NSQIP studies have biases inherent to their retrospective nature, although the database is widely accepted given its rigorous chart abstraction and quality assurance mechanisms. Randomized prospective studies have not been performed to analyze the effects of discharge destination, likely due to practical obstacles and ethical considerations surrounding the patient-specific considerations that influence discharge disposition. Finally, our Kaplan–Meier analysis showed a delay in the benefit of home discharge, as a separation was only seen after 15 days and grew over time. This trend is understandable, as discharge destination may have subacute effects on health that are slow to affect patient health. As NSQIP data are limited to 30 postoperative days, further study extending beyond this period may show a greater benefit of home discharge.

These findings have multiple systemic implications. Home discharge rates have steadily increased due to contemporary financial incentives and insurance patterns [22]. Recent studies correlate accountable care organizations and bundled payments with reduced post-acute facility placement [30]. It is widely argued that because 30-day readmissions and postoperative complications are interrelated, hospitals should not be doubly penalized by Medicare. Previous analyses have built upon this concept by showing that most postoperative complications occur due to factors that influence health after patients leave the hospital, not as exacerbations of complications from the index hospitalization [7,27,45]. Hence, policymakers must ensure that efforts to incentivize quality improvement do not inadvertently penalize hospitals for factors outside their control. Further study is required to determine situations in which hospitals are accountable for readmissions versus those outside the hospital’s control, particularly because our study indicates that post-acute care facility placement may be associated with increased readmissions.

In conclusion, our study was the first to perform rigorous statistical analyses on a large, generalizable cohort of surgical patients in order to demonstrate that non-home discharge disposition may increase the risk of unplanned readmission, various complications, and death, even after correction for poorer baseline health status. These findings indicate that quality improvement programming should be adjusted to account for extra-hospital factors to ultimately improve systemic measures and patient experiences. Further study is needed to evaluate the effects of discharge disposition beyond the near term.

## Figures and Tables

**Table 1:** Pre-discharge characteristics according to discharge destination before propensity score matching.

	Total	Discharge Destination		SMD *
		Non-Home	Home	
	*n* = 1,577,184	*n* = 194,691	*n* = 1,382,493	
Age (years), mean (SD)	62.03 (13.53)	71.19 (11.10)	60.74 (13.34)	0.852
Sex				−0.025
Female	945,997 (59.98)	118,856 (61.05)	827,141 (59.83)	
Male	631,187 (40.02)	75,835 (38.95)	555,352 (40.17)	
Race				−0.226
White	1,131,596 (71.75)	148,368 (76.21)	983,228 (71.12)	
Black	151,101 (9.58)	25,820 (13.26)	125,281 (9.06)	
Others	54,849 (3.48)	5406 (2.78)	49,443 (3.58)	
Unknown	239,638 (15.19)	15,097 (7.75)	224,541 (16.24)	
Hispanic ethnicity				−0.176
Yes	99,874 (6.33)	11,235 (5.77)	88,639 (6.41)	
No	1,249,645 (79.23)	168,856 (86.73)	1,080,789 (78.18)	
Unknown	227,665 (14.43)	14,600 (7.50)	213,065 (15.41)	
BMI				−0.040
Underweight (<18.5 kg/m^2^)	21,647 (1.37)	4495 (2.31)	17,152 (1.24)	
Normal (18.5–24.9 kg/m^2^)	317,095 (20.11)	42,227 (21.69)	274,868 (19.88)	
Overweight (25–29.9 kg/m^2^)	469,138 (29.75)	54,609 (28.05)	414,529 (29.98)	
Obesity I (30–34.9 kg/m^2^)	365,615 (23.18)	43,747 (22.47)	321,868 (23.28)	
Obesity II (35–39.9 kg/m^2^)	213,230 (13.52)	26,672 (13.70)	186,558 (13.49)	
Obesity III (≥40 kg/m^2^)	190,459 (12.08)	22,941 (11.78)	167,518 (12.12)	
Hypertension	866,165 (54.92)	141,935 (72.90)	724,230 (52.39)	0.434
Diabetes mellitus	285,369 (18.09)	53,609 (27.54)	231,760 (16.76)	0.262
Smoker within the past year	241,397 (15.31)	27,507 (14.13)	213,890 (15.47)	−0.038
ASA Status				0.575
1—No disturbance	41,055 (2.60)	974 (0.50)	40,081 (2.90)	
2—Mild disturbance	659,692 (41.83)	45,720 (23.48)	613,972 (44.41)	
3—Severe disturbance	775,482 (49.17)	116,980 (60.08)	658,502 (47.63)	
4—Life-threatening disturbance	99,346 (6.30)	30,063 (15.44)	69,283 (5.01)	
5—Moribund	1609 (0.10)	954 (0.49)	655 (0.05)	
Congestive heart failure	12,748 (0.81)	5024 (2.58)	7724 (0.56)	0.163
Chronic obstructive pulmonary disease	73,469 (4.66)	17,576 (9.03)	55,893 (4.04)	0.203
Functional status				−0.316
Independent	1,548,244 (98.17)	180,433 (92.68)	1,367,811 (98.94)	
Partially dependent	25,892 (1.64)	12,775 (6.56)	13,117 (0.95)	
Totally dependent	3048 (0.19)	1483 (0.76)	1565 (0.11)	
Ascites	3288 (0.21)	923 (0.47)	2365 (0.17)	0.053
Dyspnea				0.159
At rest	5238 (0.33)	1648 (0.85)	3590 (0.26)	
Moderate exertion	100,487 (6.37)	18,918 (9.72)	81,569 (5.90)	
No	1,471,459 (93.30)	174,125 (89.44)	1,297,334 (93.84)	
Bleeding disorder	57,871 (3.67)	16,880 (8.67)	40,991 (2.97)	0.246
Chronic steroid use	68,543 (4.35)	11,174 (5.74)	57,369 (4.15)	0.073
>10% weight loss	29,128 (1.85)	5064 (2.60)	24,064 (1.74)	0.059
Chronic kidney disease				0.533
Stage 1 (≥90 mL/min/1.73 m^2^)	605,108 (38.37)	42,920 (22.05)	562,188 (40.66)	
Stage 2 (60–89 mL/min/1.73 m^2^)	718,002 (45.52)	88,790 (45.61)	629,212 (45.51)	
Stage 3a (45–59 mL/min/1.73 m^2^)	157,670 (10.00)	31,988 (16.43)	125,682 (9.09)	
Stage 3b (30–44 mL/min/1.73 m^2^)	62,938 (3.99)	17,859 (9.17)	45,079 (3.26)	
Stage 4 (15–29 mL/min/1.73 m^2^)	18,833 (1.19)	7318 (3.76)	11,515 (0.83)	
Stage 5 (<15 mL/min/1.73 m^2^)	14,633 (0.93)	5816 (2.99)	8817 (0.64)	
Preoperative hematocrit				−0.374
<35	227,891 (14.45)	53,206 (27.33)	174,685 (12.64)	
≥35	1,349,293 (85.55)	141,485 (72.67)	1,207,808 (87.36)	
Preoperative WBC				0.194
<4 k	51,968 (3.29)	5843 (3.00)	46,125 (3.34)	
4 k–12 k	1,433,374 (90.88)	167,151 (85.85)	1,266,223 (91.59)	
≥12 k	91,842 (5.82)	21,697 (11.14)	70,145 (5.07)	
Preoperative platelets				−0.063
150 k	83,902 (5.32)	15,424 (7.92)	68,478 (4.95)	
150 k–450 k	1,452,973 (92.12)	172,503 (88.60)	1,280,470 (92.62)	
>450 k	40,309 (2.56)	6764 (3.47)	33,545 (2.43)	
Disseminated cancer	52,799 (3.35)	4929 (2.53)	47,870 (3.46)	−0.055
Surgical subspecialty				
Vascular surgery	91,995 (5.83)	28,844 (14.82)	63,151 (4.57)	0.352
General surgery	492,711 (31.24)	42,235 (21.69)	450,476 (32.58)	−0.247
Thoracic surgery	31,405 (1.99)	1699 (0.87)	29,706 (2.15)	−0.105
Urology	57,678 (3.66)	3760 (1.93)	53,918 (3.90)	−0.117
Orthopedic surgery	663,117 (42.04)	111,905 (57.48)	551,212 (39.87)	0.358
Neurosurgery	4144 (0.26)	2032 (1.04)	2112 (0.15)	0.116
Cardiac surgery	20,764 (1.32)	3282 (1.69)	17,482 (1.26)	0.035
Gynecology	215,370 (13.66)	934 (0.48)	214,436 (15.51)	−0.577
Operative time (min), median (IQR)	115 (80–182)	103 (75–160)	116 (81–185)	−0.112
Length of stay, median (IQR)	3 (1–5)	4 (3–8)	2 (1–4)	0.564
Major pre-discharge complications	31,364 (1.99)	11,887 (6.11)	19,477 (1.41)	0.249

Unless otherwise specified, categorical variables are presented as *n* (%). * An absolute SMD ≥ 0.10 indicates imbalance between groups. SD: standard deviation; IQR: interquartile range; SMD: standardized mean difference; ASA: American Society of Anesthesiologists; WBC: white blood cells.

**Table 2:** Pre-discharge characteristics according to discharge destination after propensity score matching.

	Total	Discharge Destination		SMD *
		Non-Home	Home	
	*n* = 359,770	*n* = 179,885	*n* = 179,885	
Age (years), mean (SD)	70.31 ± 10.99	70.82 ± 11.05	69.80 ± 10.90	0.092
Sex				0.013
Female	221,251 (61.50)	110,067 (61.19)	111,184 (61.81)	
Male	138,519 (38.50)	69,818 (38.81)	68,701 (38.19)	
Race				−0.024
White	275,247 (76.51)	138,080 (76.76)	137,167 (76.25)	
Black	44,272 (12.31)	22,480 (12.50)	21,792 (12.11)	
Others	10,351 (2.88)	5034 (2.80)	5317 (2.96)	
Unknown	29,900 (8.31)	14,291 (7.94)	15,609 (8.68)	
Hispanic ethnicity				−0.013
Yes	20,798 (5.78)	10,256 (5.70)	10,542 (5.86)	
No	309,984 (86.16)	155,725 (86.57)	154,259 (85.75)	
Unknown	28,988 (8.06)	13,904 (7.73)	15,084 (8.39)	
BMI				−0.013
Underweight (<18.5 kg/m^2^)	7269 (2.02)	3756 (2.09)	3513 (1.95)	
Normal (18.5–24.9 kg/m^2^)	74,501 (20.71)	37,700 (20.96)	36,801 (20.46)	
Overweight (25–29.9 kg/m^2^)	101,591 (28.24)	50,624 (28.14)	50,967 (28.33)	
Obesity I (30–34.9 kg/m^2^)	82,427 (22.91)	41,115 (22.86)	41,312 (22.97)	
Obesity II (35–39.9 kg/m^2^)	50,602 (14.07)	25,193 (14.01)	25,409 (14.13)	
Obesity III (≥40 kg/m^2^)	43,380 (12.06)	21,497 (11.95)	21,883 (12.16)	
Hypertension	256,649 (71.34)	129,786 (72.15)	126,863 (70.52)	0.036
Diabetes mellitus	93,248 (25.92)	47,601 (26.46)	45,647 (25.38)	0.025
Smoker within the past year	50,483 (14.03)	25,160 (13.99)	25,323 (14.08)	−0.003
ASA Status				0.063
1—No disturbance	2403 (0.67)	966 (0.54)	1437 (0.80)	
2—Mild disturbance	93,323 (25.94)	45,216 (25.14)	48,107 (26.74)	
3—Severe disturbance	218,046 (60.61)	109,150 (60.68)	108,896 (60.54)	
4—Life-threatening disturbance	45,012 (12.51)	23,953 (13.32)	21,059 (11.71)	
5—Moribund	986 (0.27)	600 (0.33)	386 (0.21)	
Congestive heart failure	6928 (1.93)	3805 (2.12)	3123 (1.74)	0.028
Chronic obstructive pulmonary disease	29,689 (8.25)	15,222 (8.46)	14,467 (8.04)	0.015
Functional status				−0.051
Independent	341,278 (94.86)	169,626 (94.30)	171,652 (95.42)	
Partially dependent	16,614 (4.62)	9271 (5.15)	7343 (4.08)	
Totally dependent	1878 (0.52)	988 (0.55)	890 (0.49)	
Ascites	1384 (0.38)	734 (0.41)	650 (0.36)	0.008
Dyspnea				0.012
At rest	2315 (0.64)	1340 (0.74)	975 (0.54)	
Moderate exertion	33,498 (9.31)	16,879 (9.38)	16,619 (9.24)	
No	323,957 (90.05)	161,666 (89.87)	162,291 (90.22)	
Bleeding disorder	26,270 (7.30)	13,843 (7.70)	12,427 (6.91)	0.030
Chronic steroid use	19,492 (5.42)	9892 (5.50)	9600 (5.34)	0.007
>10% weight loss	8384 (2.33)	4313 (2.40)	4071 (2.26)	0.009
Chronic kidney disease				0.056
Stage 1 (≥90 mL/min/1.73 m^2^)	84,879 (23.59)	40,929 (22.75)	43,950 (24.43)	
Stage 2 (60–89 mL/min/1.73 m^2^)	168,790 (46.92)	84,371 (46.90)	84,419 (46.93)	
Stage 3a (45–59 mL/min/1.73 m^2^)	58,081 (16.14)	29,286 (16.28)	28,795 (16.01)	
Stage 3b (30–44 mL/min/1.73 m^2^)	30,014 (8.34)	15,440 (8.58)	14,574 (8.10)	
Stage 4 (15–29 mL/min/1.73 m^2^)	10,467 (2.91)	5656 (3.14)	4811 (2.67)	
Stage 5 (<15 mL/min/1.73 m^2^)	7539 (2.10)	4203 (2.34)	3336 (1.85)	
Preoperative hematocrit				−0.041
<35	85,121 (23.66)	44,138 (24.54)	40,983 (22.78)	
≥35	274,649 (76.34)	135,747 (75.46)	138,902 (77.22)	
Preoperative WBC				0.033
<4 k	10,786 (3.00)	5409 (3.01)	5377 (2.99)	
4 k–12 k	316,457 (87.96)	157,175 (87.38)	159,282 (88.55)	
≥12 k	32,527 (9.04)	17,301 (9.62)	15,226 (8.46)	
Preoperative platelets				−0.006
150 k	26,489 (7.36)	13,579 (7.55)	12,910 (7.18)	
150 k–450 k	322,105 (89.53)	160,557 (89.26)	161,548 (89.81)	
>450 k	11,176 (3.11)	5749 (3.20)	5427 (3.02)	
Disseminated cancer	9157 (2.55)	4452 (2.47)	4705 (2.62)	−0.009
Surgical subspecialty				−0.061
Vascular surgery	44,260 (12.30)	23,394 (13.00)	20,866 (11.60)	
General surgery	75,861 (21.09)	37,906 (21.07)	37,955 (21.10)	
Thoracic surgery	3364 (0.94)	1623 (0.90)	1741 (0.97)	
Urology	7313 (2.03)	3562 (1.98)	3751 (2.09)	
Orthopedic surgery	215,565 (59.92)	108,091 (60.09)	107,474 (59.75)	
Neurosurgery	2374 (0.66)	1378 (0.77)	996 (0.55)	
Cardiac surgery	5869 (1.63)	3000 (1.67)	2869 (1.59)	
Gynecology	5164 (1.44)	931 (0.52)	4233 (2.35)	
Operative time (min), median (IQR)	102 (75–159)	103 (75–158)	101 (74–161)	−0.011
Length of stay, median (IQR)	3 (2–7)	4 (3–7)	3 (2–6)	0.106
Major pre-discharge complications	15,766 (4.38)	8755 (4.87)	7011 (3.90)	0.047

Unless otherwise specified, categorical variables are presented as *n* (%). * An absolute SMD ≥ 0.10 indicates imbalance between groups. Propensity score matching was based on a logistic regression model. The model included all variables listed in the table (92.4% match rate = 179,885/194,691 cases matched). SD: standard deviation; IQR: interquartile range; SMD: standardized mean difference; ASA: American Society of Anesthesiologists; WBC: white blood cells.

**Table 3:** Rates of unplanned readmission and post-discharge complications according to discharge destination after propensity score matching.

Outcome	Discharge Destination		*p*-Value	OR (95% CI) ^a^ Ref: Home Discharge	E-Value ^b^ (Effect Estimate)	E-Value ^b^ (CI Limit)
	Non-Home (*n* = 179,885)	Home (*n* = 179,885)				
Unplanned readmission	16,649 (9.26)	13,209 (7.34)	<0.001	1.27 (1.23–1.30)	1.85	1.77
Post-discharge pulmonary complications	894 (0.50)	338 (0.19)	<0.001	2.63 (2.33–3.03)	4.70	4.08
Post-discharge infectious complications	9596 (5.33)	6970 (3.87)	<0.001	1.37 (1.32–1.41)	2.08	1.96
Post-discharge venous thromboembolism	1822 (1.01)	1204 (0.67)	<0.001	1.52 (1.41–1.61)	2.40	2.17
Post-discharge bleeding requiring transfusion	882 (0.49)	435 (0.24)	<0.001	2.56 (2.22–2.94)	4.57	3.87
Death	1845 (1.03)	773 (0.43)	<0.001	2.38 (2.17–2.63)	4.19	3.77

^a^ Obtained from a conditional logistic model after adjusting for length of stay (unbalanced between the two groups after PSM) and accounting for the clustering created through matching. ^b^ E-values for effect measures and CI limits for the associations between non-home discharge and the outcomes of interest.

## Data Availability

The data are not publicly available due to legal restrictions.

## Supplementary Materials

The following supporting information can be downloaded at: https://www.mdpi.com/article/10.3390/jcm12216784/s1, Table S1: List of CPT codes for the multiprocedural cohort.
