# Supplementary material for: Postoperative Discharge Destination Impacts 30-Day Outcomes: A National Surgical Quality Improvement Program Multi-Specialty Surgical Cohort Analysis
Source: J Clin Med. 2023 Oct 26;12(21):6784. doi: 10.3390/jcm12216784 (PMC10650337; doi:10.3390/jcm12216784)
Supplement: Supplementary file 1 [file jcm-12-06784-s001.zip › jcm-2633277-supplementary.pdf]

**Supplementary Table S1.** List of CPT codes for the multiprocedural cohort

| **Description** | **CPT codes** |
|---|---|
| Vascular Surgery | |
| Open abdominal aortic aneurysm repair | 34830, 34831, 34832, 35081, 35082, 35091, 35092, 35102, 35103 |
| Endovascular abdominal aortic aneurysm repair | 34701, 34702, 34703, 34704, 34705, 34706 |
| Below knee amputation | 27880, 27882, 27881 |
| Above knee amputation | 27592, 27590, 27591 |
| Peripheral vascular surgery<br><br>(Artery and vein bypass procedures: aorto-bifemoral, aorto-femoral, ileo-femoral, femoral-popliteal, femoral-tibial, aorto-femoral-popliteal, femoral-femoral) | 35551, 35646, 35647, 35666, 35665, 35540, 35539, 35566, 35565, 35585, 35656, 35556, 35583, 35548, 35661, 35558 |
| General Surgery | |
| Large bowel/rectal surgery | 44150, 44151, 44155, 44156, 44157, 44158, 44211, 44212, 44210, 44120, 44204, 44206, 44207, 44208, 44140, 44141, 44143, 44144, 44145, 44146, 45110, 45112, 45119, 45120, 45121, 45395, 45397 |
| Hepatic/Liver resection | 47379, 47120, 47122, 47125, 47130 |
| Whipple's pancreaticoduodenectomy | 48999, 48150, 48152, 48153, 48154 |
| Gastric bypass | 43846, 43847, 43644r, 43645 |
| Thoracic surgery | |
| Open pneumonectomy or lobectomy | 32440, 32442, 32445, 32488, 32482, 32480, 32484, 32486, 32491 |
| VATS lobectomy | 32491T, 32663 |
| Urology | |
| Radical Cystectomy | 51570, 51590, 51575, 51596, 51580, 51570, 51585, 51595 |
| Nephrectomy | 50225, 50230, 50220, 50545, 50546 |
| Nephroureterectomy | 50234, 50236 |
| Orthopedic Surgery | |
| Total hip and knee joint replacement | 27130, 27447, 27445 |
| Spinal surgery (Multisegment surgery, cervical arthroplasty/replacement) | 22845 22846 22847 |

| | |
|---|---|
| | 22856 22858<br><br>22840 22841 22842 22843 22844<br><br>63050 63051<br><br>22845 22846 22847<br><br>22845 22846 22847<br><br>22840 22841 22842 22843 22844<br><br>22840 22841 22842 22843 22844<br><br>22840 22841 22842 22843 22844 |
| Hip fixation | 27253, 27254, 27259 |
| Neurosurgery | |
| Open craniotomy, craniectomy, posterior fossa surgery[3] | 61304, 61305, 61522, 61312, 61313, 61314, 61315, 61322, 61323 |
| Cardiac Surgery | |
| Coronary artery bypass grafting | 33510, 33511, 33512, 33514, 33515, 33516, 33517, 33518, 33519, 33520, 33521, 33522, 33523, 33533, 33534, 33535, 33536 |
| Single valve repair or replacement[4] | 33602, 33391, 33390, 33600, 33425, 33426, 33427, 33420, 33422, 33470, 33471, 33474, 33468, 33463, 33464, 33430, 33465, 33475, 33405, 33406, 33410 |
| Complex: multiple valve surgeries, aortic procedures, ventricular aneurysmectomy, assist device[5] | 33866, 33863, 33413, 33440, 33412, 33411, 33548, 33542, 33975, 33979, 33622 |
| Gynecology | |
| Hysterectomy | 58152, 58541-58544, 58260-58294, 58570, 58571, 58572, 58573, 58550, 58552, 58553, 58554, 58542, 58541, 58543, 58544 |
